# A Novel Polishing Paste (Mollusk Shells) for Poly (Methylmethacrylate)

**DOI:** 10.1155/2021/5511797

**Published:** 2021-07-03

**Authors:** Nicole Guerra, Evelin Meneses, Stefany Caballero-García, Frank Mayta-Tovalino

**Affiliations:** ^1^School of Dentistry, Faculty of Health Sciences, Universidad Peruana de Ciencias Aplicadas, Lima, Peru; ^2^Posgraduate Department, CHANGE Research Working Group, Faculty of Health Sciences, Universidad Cientifica del Sur, Lima, Peru

## Abstract

**Objective:**

The aim of this study was to evaluate the effectiveness of a mollusk shells polishing paste (*Donax obesulus*) on the surface roughness of acrylic resin poly (methylmethacrylate) (PMMA).

**Methods:**

This study was an in vitro experimental design. A sample size of 72 was divided into 4 groups of *n* = 18 each. PMMA specimens were prepared and polished with the evaluated pastes using mollusk shells (experimental paste) and pumice stone. Surface roughness (*μ*m) was measured using a profilometer after polishing the PMMA samples. The paired Wilcoxon test was used to evaluate the roughness values at 24 and 48 hours. Then, the Mann–Whitney *U* test was used to identify the differences between the effects of the two groups evaluated with a significance level of *α* = 0.05.

**Results:**

The roughness difference between the pastes under study was compared, and mean values of 0.50 ± 0.07 *μ*m (mollusk shell paste group) and 0.45 ± 0.12 *μ*m (pumice group) were obtained. No statistically significant differences were found between the experimental paste and pumice stone paste (*p*=0.309). The specimens polished with pumice stone paste showed higher roughness values, while those polished with the experimental paste exhibited the lowest values*. Conclusion*. In summary, mollusk shells polishing paste had a decrease in roughness values compared to pumice, although these differences were not statistically significant.

## 1. Introduction

Poly (methylmethacrylate) (PMMA) acrylic resin is the material of choice in prosthetic treatments because it is a reliable, low-cost biomaterial with acceptable physicochemical and esthetic properties. However, these properties are affected by different factors (inadequate polishing or its absence), which generate irregularities in their surfaces [1–3]. Surface roughness of acrylics used in dental prostheses is an important criterion, and studies support that a rough surface leads to the retention of bacterial plaque and, consequently, diminishes its clinical success [4–10]. In vivo studies have shown that values above the 0.2 *μ*m threshold for acrylic resins contribute to an increase in bacterial plaque in the oral cavity [5, 11–16]. Using polishing pastes with adequate abrasive properties is recommended for the polishing of acrylic resins to reduce roughness [10, 17].

Despite the variety of pastes available in the market, there is a demand for natural products, especially those with marine origins, which allow us to have supplies used in dental clinical practice [18]. Therefore, some studies have evaluated mollusk shells for their high mechanical properties and abrasive components, such as calcium carbonate. Khartic et al. [15] and Morris et al. [16] evaluated the shells of mollusks for the elaboration of new materials useful in dentistry, such as for the reinforcement of dental prostheses and for the increase of the mechanical properties of the fluid resins [9] to find favorable results. Despite the various investigations on mollusk shells, no studies evaluating the surface roughness with experimental pastes made using mollusk shells have been reported. Therefore, it is necessary to study the properties of mollusk shells and evaluate the surface roughness of acrylic resins since adequate roughness is a fundamental parameter for its longevity, esthetics, and treatment success [2–4].

The main factors that must be controlled by polishing are reducing the adherence of the biofilm, facilitating the hygiene of dental prostheses, and guaranteeing a greater longevity of the restorations [6–11]. The polishing of the acrylics is a very important factor because this allows a correct elimination of the microroughness of the acrylic surfaces. Currently, there are various polishing materials such as silicone rubbers and pumice; however, polishing pastes also play an essential role in achieving homogenization of the acrylic surface of dental prostheses [19].

The importance of this study consists in contributing to the environmental contribution, giving a second utility to the different natural resources that we find on the planet. For this reason, the creation of this mollusk shell polishing paste is proposed as an alternative to pumice. Above all, ensure adequate availability of an easily accessible and low-cost polishing paste. We hypothesized that the experimental mollusk shell paste could reduce the surface roughness of the acrylic resin to lower than the acceptable range of 0.2 *μ*m. Thus, in this study, we compared the effectiveness of mollusk shell paste (*Donax obesulus*) on the surface roughness of PMMA in vitro.

## 2. Materials and Methods

### 2.1. Sample Size and Study Design

This study used an in vitro experimental design. The sample size was calculated by the means comparison formula using Statistical Software Stata® 15, with an alpha of 0.05 and a test power of 0.80. Finally, a sample size of 72 was divided into 4 groups of *n* = 18 each.

The following groups were formed:

  Group 1: PMMA blocks initially polished with mollusk shell paste
  Group 2: PMMA blocks initially polished with pumice
  Group 3: PMMA blocks polished after 24 hours with mollusk shell paste
  Group 4: PMMA blocks polished after 24 hours with pumice

### 2.2. Preparation of Experimental Paste Based on Mollusk Shells

**Figure 1:**
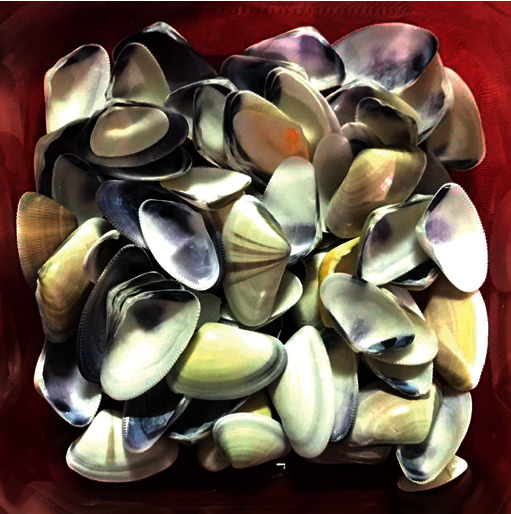
Collection and selection of mollusk shells (*Donax obesulus*).

**Figure 2:**
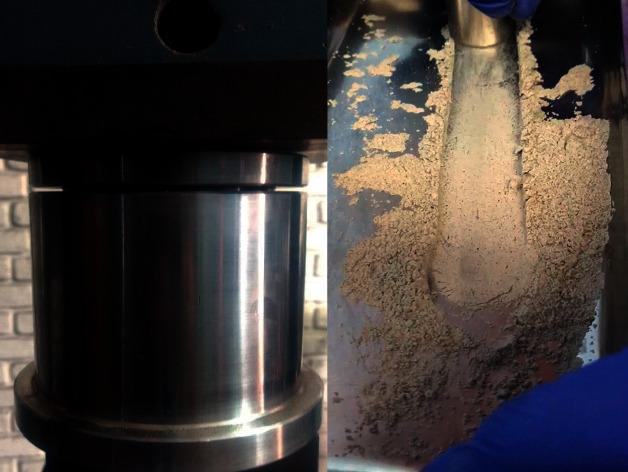
Pulverized mollusk shells.

*Donax obesulus* (250 g) was collected with the following characteristics: similar-sized convex valves, with rounded ends, trapezoidal profile, and a brown-yellowish color (Figure 1). They were then brought to a boiling point for 5 min, washed with distilled water, and disinfected with a dilute solution of 4% sodium hypochlorite for 6 h at room temperature (15–30°C). They were then vacuum dried for 8 min at 250°C and then crushed with a WEW-300B digital hydraulic press (Liangong, China) (Figure 2). It exerted a force of 1 ton (9806.65 N) on them for 2 min for compression. Subsequently, manual crushing was carried out with stainless steel bodies for 5 min to obtain small particles. The powders were placed on a 95, 45, and 15 *μ*m sieve (Standard Testing Sieve, USA) to segregate particles on the basis of size; according to specification no. 37, the powder was sieved to obtain medium caliber powders (15 mm) [6].

### 2.3. Preparation of PMMA Specimens

**Figure 3:**
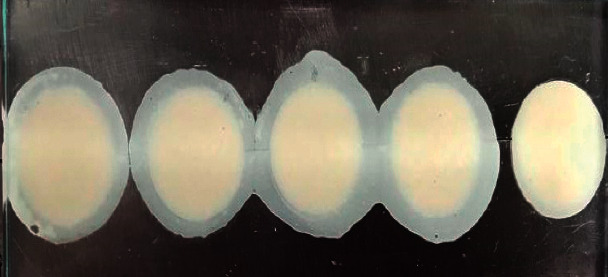
Autopolymerization of acrylic specimens.

A metallic stainless steel matrix (140 × 50 × 6 cm) divided into five holes (20 × 6 mm) was used to make specimens of PMMA (Duralay temporary crown and bridge kit, code 41000000, Reliance Dental Manufacturing LLC, Illinois, USA) (Figure 3). The metal matrix and two glass plates (125 × 50 × 5 mm) were isolated to avoid adhesion of the bodies to them using a brush covered with liquid Vaseline. All the specimens were autopolymerized according to the manufacturer's instructions. For the preparation of PMMA, 2 parts of powder were mixed with one part of liquid. After, excess removal of the acrylic blocks was carried out with a medium-grain tungsten carbide bur (C21L, Code P.72, Jota®, Hirschensprung, Switzerland) at 1500 rpm.

### 2.4. Polishing of PMMA Specimens

**Figure 4:**
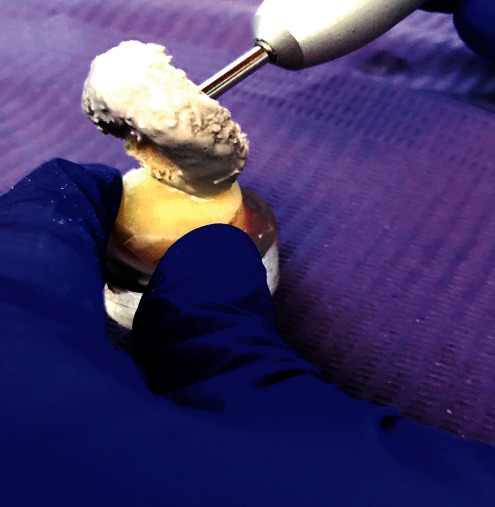
Polishing of polymethylmethacrylate blocks.

The specimens were fixed on acrylic bases 30 mm in diameter and 12 mm in height with pink wax (Cavex, Code WA005, Haarlem, Netherlands). Upon completion, the specimens were stored in a wet glass container. A micromotor (NSK Ti-Max X205L, Japan) and three rag wheels (Code 1164, Jota®, Hirschensprung, Switzerland), one wheel for every specimen, was used for polishing with the experimental paste. The powder and distilled water were mixed for every specimen to complete all the blocks. Prior to polishing, half of the specimens were covered with a nitrile fragment to differentiate the initial and final roughness of each specimen and prevent contamination because same specimens were used for initial and final roughness. The polishing was performed in a straight movement from left to right at a speed of 1500 rpm for 2 min (Figure 4). The procedures were performed by a single operator exerting the same pressure to eliminate the difference in polishing between the specimens. The force exerted was similar to that exerted by gentle tooth brushing (2 N). In the same way, the same procedure was carried out for polishing PMMA blocks with pumice stone paste, with the measures mentioned above. The specimens were washed with distilled water for 10 s and air dried using a triple syringe for 8 s to remove polishing paste residues. They were then stored in a container with distilled water in an oven for 24 h at 37 ±°5°C.

### 2.5. Analysis of Surface Roughness

**Figure 5:**
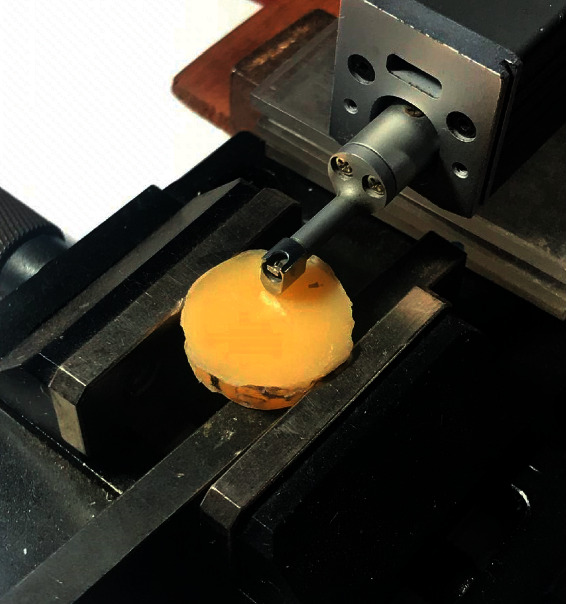
Roughness test.

The surface roughness of the PMMA specimens was analyzed using a previously calibrated Mitutoyo roughness meter (Surftest SJ-210, Kanagawa, Japan). The roughness of each specimen was measured using the digital needle of the profilometer, located in three different positions, with a distance of 0.25 mm between each reading (constant velocity of 0.1 mm/s, force of 0.7 N, and radius of 1.5 *μ*m). The arithmetic mean of each of the three measurements was obtained to estimate the initial and final roughness of each specimen in micrometers (*μ*m) (Figure 5).

### 2.6. Statistical Analysis

For the descriptive statistics, the arithmetic means, standard deviations, medians, and interquartile ranges were obtained using the Stata® 15 software, and the Shapiro–Wilk test was used to determine the normality of the values of surface roughness in *μ*m. The results of the initial and final roughness of the two study pastes, using the paired Wilcoxon test and the Mann–Whitney *U* test, were used to find a statistically significant difference (*α* = 0.05).

## 3. Results

### 3.1. In Vitro Evaluation of Surface Microroughness

It was found that none of the groups 1, 2, 3, and 4 had a normal distribution. In the basal measurement, the mollusk shell paste had a roughness of 0.56 ± 0.07 *μ*m, while the pumice stone had 0.61 ± 0.1 *μ*m. However, roughness decreased at 24 hours; in the group of specimens that were polished with mollusk shell paste, they had 0.09 ± 0.03 *μ*m; while in the pumice group, 0.16 ± 0.06 *μ*m was found. A statistically significant difference between the initial and final roughness of the experimental paste was observed; similarly, the control group yielded the same results between both roughness values (*p*=0.001) (Table 1).

Likewise, the roughness difference between the pastes under study was compared, and the mean values of 0.50 ± 0.07 *μ*m and 0.45 ± 0.12 *μ*m were obtained. No statistically significant difference was observed between the surface roughness of the experimental paste and the control groups (*p*=0.309).

## 4. Discussion

The present study evaluated the efficacy of an experimental mollusk shell paste for polishing PMMA surfaces and compared it with that of pumice stone paste. The hypothesis raised in the research was accepted based on the results of this study, which showed that there was a significant decrease in the surface roughness of the PMMA specimens that were polished with the experimental paste (*p* < 0.001). Finally, it was decided to evaluate the roughness of the PMMA blocks at 24 hours because it is the time in which self-cured acrylics generally reach their maximum shrinkage peak.

The roughness values found in the present study indicate that the PMMA surfaces polished with the experimental paste and pumice stone paste were within the acceptable range of surface roughness in the oral cavity (0.2 *μ*m) [5–7]. Therefore, it can be inferred that this new mollusk shell-based paste could be used as an input in dentistry because it is effective in reducing the surface roughness of acrylic resins.

One of the factors that could be related to the decrease in the surface roughness of PMMA may be the particle size of the mollusk shells (15 *μ*m), which is of medium size according to the American Dental Association. This supports the argument of some authors who claim that finer particle abrasive materials reduce surface roughness more effectively [6, 12]. Another factor that could influence the decrease in surface roughness found in this study can be attributed to the chemical composition of the mollusk shells, which are composed mostly of CaCO_3_. Various authors affirm that this component provides abrasive properties that generate erosion by eliminating extrinsic stains. Therefore, it is believed that this biomaterial could generate less roughness on acrylic surfaces [9, 11].

The findings of the present study show a roughness of 0.09 *μ*m compared to that of the control group, obtaining similar results with most of the polishing pastes currently in the market. These results were similar to those obtained in the study by Rao, who evaluated the roughness of acrylic resins polished with a universal paste and pumice stone paste and concluded that all the pastes analyzed decreased the roughness values. Similarly, various investigations that evaluated this property with commercial polishing pastes frequently used in dentistry have obtained similar results to those of the present study [6, 12–17].

The results of this investigation revealed that the 15 *μ*m experimental paste (Ra = 0.09 *μ*m) produced a better polish on PMMA surfaces than the 15 *μ*m pumice stone paste (Ra = 0.16 *μ*m); however, the difference was not statistically significant (*p* < 0.001). This subject has been scarcely studied in dentistry, and the evaluation of surface roughness using the experimental paste is limited in the literature. Therefore, this line of research is expected to continue to test new experimental compounds, since there is a great deal of work being done currently. In addition, with regards to the effects of using mollusk shell pastes, there have been reports earlier [12–15].

The main limitation of the present study is that there was no control over the force exerted during the acrylic polishing; in addition, there was an absence of a device that could allow its measurement. However, certain criteria were considered, such as the choice of a single operator to perform the polishing and taking short breaks after every five acrylic bodies to avoid fatigue for the operator. Another limitation was that only the microroughness of self-curing PMMA was evaluated because it is the material indicated for a rapid fabrication of temporary crowns. Nonetheless, the present study offers a new ecological alternative to polishing paste for acrylic resins. By obtaining favorable results in reducing surface roughness, new information and options for polishing pastes are being brought to the market of the dental field. Finally, this research can be the basis of future studies to evaluate the different properties of the experimental paste made from mollusk shells. It is recommended to continue this line of research with state-of-the-art instruments such as electron microscopy that allow deepening the mechanism of action of the particles of the mollusk shell polishing paste.

## 5. Conclusions

In conclusion, the experimental mollusk shell-based paste reduced the surface roughness values on the surfaces of dental acrylic resins. This suggests that the mollusk shell particle size used in the experimental paste provided clinically acceptable polished surfaces.

## Figures and Tables

**Table 1:** In vitro evaluation of the surface roughness of the experimental mollusk shell paste versus pumice.

Paste	Initial		24 hours				Mean difference		
	Mean ± SD (*μ*m)	Median (*μ*m)	Mean ± SD (*μ*m)	Median (*μ*m)	*P* ^*∗*^	*P* ^‡^	Mean ± SD (*μ*m)	Median (*μ*m)	*P* ^†^
Mollusk shell paste	0.56 ± 0.07	0.54	0.09 ± 0.03	0.08	<0.05	<0.001	0.50 ± 0.07	0.43	0.309
Pumice	0.61 ± 0.11	0.58	0.16 ± 0.06	0.15	<0.05	<0.001	0.45 ± 0.12	0.46	

^*∗*^Normality test (Shapiro–Wilk, *p* < 0.05). ^‡^Paired Wilcoxon test. ^†^Mann–Whitney *U* test. Significance level, *p* < 0.05.

## Data Availability

The datasets that support the findings of this study are available from the corresponding author upon request.
